# Metabolic capability and in situ activity of microorganisms in an oil reservoir

**DOI:** 10.1186/s40168-017-0392-1

**Published:** 2018-01-05

**Authors:** Yi-Fan Liu, Daniela Domingos Galzerani, Serge Maurice Mbadinga, Livia S. Zaramela, Ji-Dong Gu, Bo-Zhong Mu, Karsten Zengler

**Affiliations:** 1 State Key Laboratory of Bioreactor Engineering and Institute of Applied Chemistry, East China University of Science and Technology, 130 Meilong Road, Xuhui District, Shanghai, 200237 People’s Republic of China; 2 Department of Pediatrics, University of California, San Diego, 9500 Gilman Drive, La Jolla, San Diego, CA 92093-0760 USA; 3 School of Biological Sciences, The University of Hong Kong, Pokfulam Road, Hong Kong, People’s Republic of China; 4 Shanghai Collaborative Innovation Center for Biomanufacturing Technology, Shanghai, 200237 People’s Republic of China; 5 Center for Microbiome Innovation, University of California, San Diego, 9500 Gilman Drive, La Jolla, San Diego, CA 92093-0436 USA

**Keywords:** Auxotrophy, Hydrocarbon degradation, Metagenomics and metatranscriptomics, Microbial community, Oil reservoir

## Abstract

**Background:**

Microorganisms have long been associated with oxic and anoxic degradation of hydrocarbons in oil reservoirs and oil production facilities. While we can readily determine the abundance of microorganisms in the reservoir and study their activity in the laboratory, it has been challenging to resolve what microbes are actively participating in crude oil degradation in situ and to gain insight into what metabolic pathways they deploy.

**Results:**

Here, we describe the metabolic potential and in situ activity of microbial communities obtained from the Jiangsu Oil Reservoir (China) by an integrated metagenomics and metatranscriptomics approach. Almost complete genome sequences obtained by differential binning highlight the distinct capability of different community members to degrade hydrocarbons under oxic or anoxic condition. Transcriptomic data delineate active members of the community and give insights that *Acinetobacter* species completely oxidize alkanes into carbon dioxide with the involvement of oxygen, and *Archaeoglobus* species mainly ferment alkanes to generate acetate which could be consumed by *Methanosaeta* species. Furthermore, nutritional requirements based on amino acid and vitamin auxotrophies suggest a complex network of interactions and dependencies among active community members that go beyond classical syntrophic exchanges; this network defines community composition and microbial ecology in oil reservoirs undergoing secondary recovery.

**Conclusion:**

Our data expand current knowledge of the metabolic potential and role in hydrocarbon metabolism of individual members of thermophilic microbial communities from an oil reservoir. The study also reveals potential metabolic exchanges based on vitamin and amino acid auxotrophies indicating the presence of complex network of interactions between microbial taxa within the community.

**Electronic supplementary material:**

The online version of this article (10.1186/s40168-017-0392-1) contains supplementary material, which is available to authorized users.

## Background

Microorganisms have been detected in oil reservoirs, pipelines, and crude oil processing facilities for decades [1, 2]. Their presence has long been associated with their metabolic capability of degrading crude oil hydrocarbons, under both oxic and anoxic conditions [3, 4]. While oil reservoirs are generally considered anoxic, molecular oxygen present in water injected into the reservoir during secondary recovery can play an important role as electron acceptor and can facilitate activation of otherwise chemically inert hydrocarbons [5]. Microorganisms associated to taxa consisting of predominately aerobic members have been detected and associated to the degradation of hydrocarbon in oil reservoirs undergoing secondary recovery [6].

Over the past decades, we have gained in depth knowledge, mainly through laboratory experiments, about how hydrocarbons are metabolized under oxic and anoxic conditions [7, 8]. Furthermore, recent advances into molecular biology and metabolomics have enabled elucidation of the metabolic potential of microbial communities existing in oil reservoirs [9]. Currently, however, we lack a deeper understanding of which members of the community are active in oil reservoirs and if these organisms are contributing to the degradation of crude oil in situ concurrently. Here, we analyze the aerobic and anaerobic microbial communities obtained from three wells of the Jiangsu Oil Reservoir (China) by an integrated metagenomic and metatranscriptomic approach to gain insight into the activity of specific microbes in situ. We annotate (almost complete) genomes from these samples and determine different activation mechanisms deployed during hydrocarbon degradation*.* Furthermore, we reveal nutritional requirements of oil reservoir microorganisms, such as amino acids and vitamins, hinting at a complex network of microbial interactions beyond syntrophy present in oil reservoirs.

## Results

### Metagenomic analysis of oil reservoir communities

**Fig. 1:**
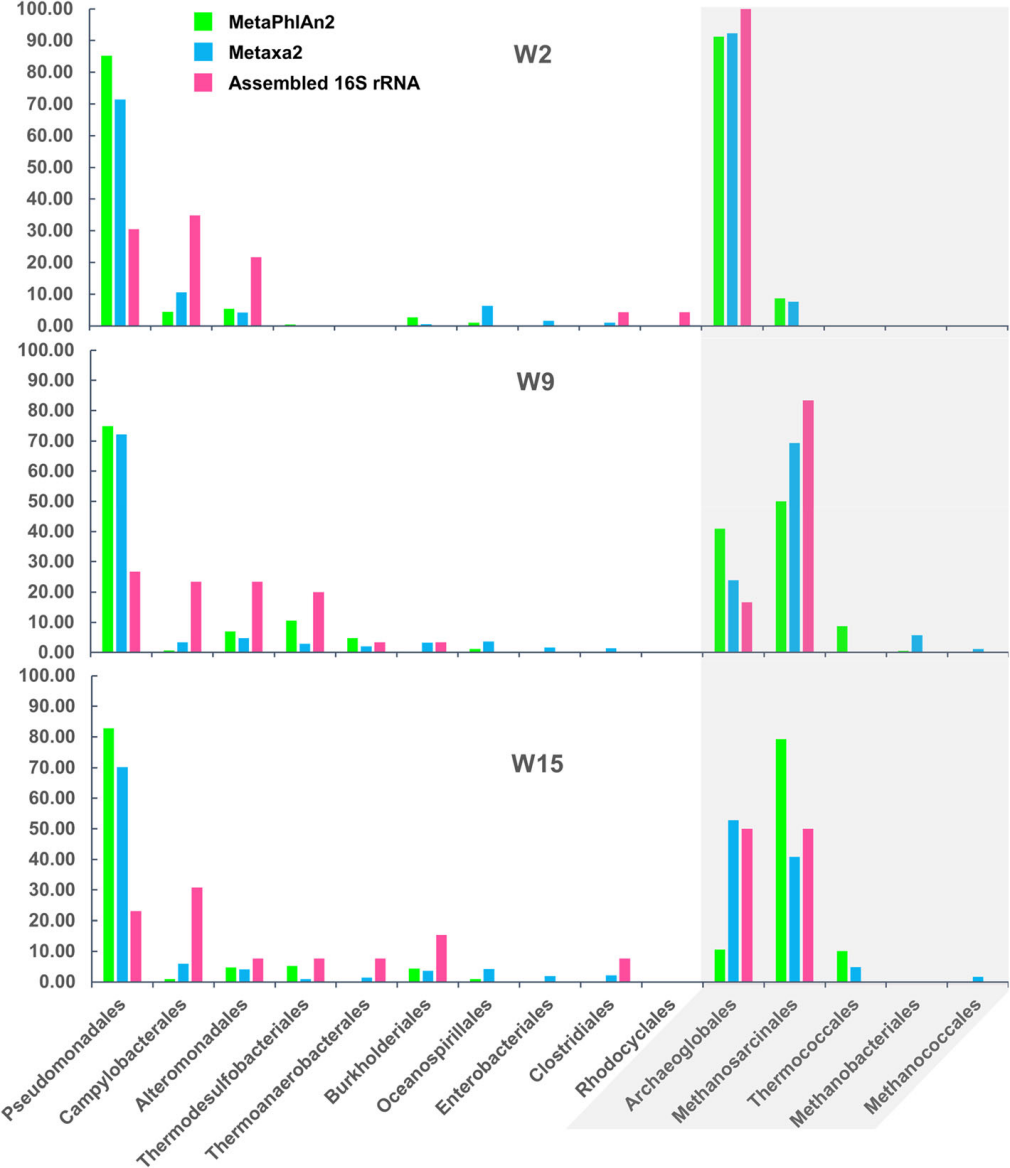
Taxonomic analysis of metagenomes evaluated from unassembled reads by MetaPhlAn2 and Metaxa2 and from assembled 16S rRNA gene sequences. Bar graph is showing the percent abundance of the different members at order level, and only members with more than 0.1% abundance in either sample is showed here. Archaeal members are marked in gray. To get a better visualization, relative abundances of archaeal members are normalized to total number of archaeal members

Shotgun metagenomic sequencing of DNA obtained from wells W2-71, W9-18, and W15-5 in the Jiangsu Oil Reservoir (Jiangsu, China) generated ~ 9,200,000, ~ 10,900,000, and ~ 7,700,000 quality-controlled paired-end reads (2 × 75 bp), respectively (see Additional file 1: Table S1). In the remainder of this article, the three samples will be denoted as W2, W9 and W15. Sequence coverage was high for all three samples, ranging from 80 to 94% completion (see Additional file 2: Table S2) as estimated by Nonpareil [10]. Taxonomic classification from unassembled reads using MetaPhlAn2 and Metaxa2 yielded improved resolution over the assembled 16S rRNA gene sequences (Fig. 1). The biased estimation of taxonomic diversity is probably due to the low number of OTUs (13/17/16 (W2/W9/W15) clustered from 39/39/25 16S rRNA gene sequences) that were obtained from separately assembled contigs of the three samples (see Additional file 3: Table S3). As highlighted in Fig. 1, the most abundant bacterial orders in all three samples were *Pseudomonadales*, followed by *Alteromonadales*, *Campylobacterales*, and *Thermodesulfobacteriales* (Fig. 1). MetaPhlAn2 analysis revealed different proportions of Archaea compared to Metaxa2. This disaccord is expected since different biomarkers are used by these two tools to evaluate taxonomic information from metagenomes. Archaea sequences were dominated by members of the *Methanosarcinales* and *Archaeoglobales*. The overall microbial composition of three samples are similar except that members of *Thermodesulfobacteriales* and *Thermoanaerobacterales* were not detected in W2 samples, and relative low abundance of *Methanosarcinales* was found in W2 compared to other two samples. This is probably due to the lowest sequence coverage of W2 among the three samples, which affects abundance of minor members in microbial community, such as Archaeal members and minor community members of Bacteria. This is supported by a recent study in which similar microbial composition was reported in the same wells (W2-71 and W9-18) [11].

Metagenomic datasets were used to carry out differential coverage binning. A total of 44 genome bins (GBs) across all three samples were recovered (see Additional file 4: Table S4), representing > 95% of OTUs recovered (see Additional file 3: Table S3). Raw reads of the metagenome datasets were mapped to the GBs and normalized based on genome size to determine their relative abundance in each sample, and the result coincided with the result of taxonomic classification analysis above (see Additional file 4: Table S4). Seven high-quality genomes (completeness > 85%, contamination < 3%) namely *Acinetobacter*-like Bin1, *Marinobacter*-like Bin13, *Sulfurimonas*-like Bin29, *Thermodesulfobacterium*-like Bin7, *Archaeoglobus*-like Bin9, *Archaeoglobus*-like Bin16, and *Methanosaeta*-like Bin39, which represent the most dominant members of *Pseudomonadales*, *Alteromonadales*, *Campylobacterales*, *Thermodesulfobacteriales*, *Archaeoglobales* and *Methanosarcinales* identified by assembled 16S rRNA gene sequences (Fig. 1, Additional file 3: Table S3).

### Metabolic potential in genome bins

**Fig. 2:**
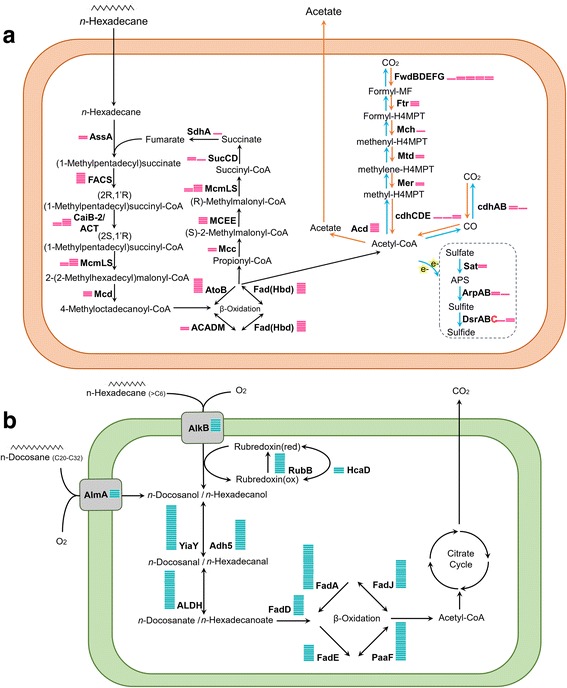
Metabolic reconstruction of a putative model for *n*-hexadecane degradation in Bin9 (*Archaeoglobus*) and Bin1 (*Acinetobacter*). Expression level of each gene is represented by bars, one bar equaling a FPKM value of 10. Genes whose FPKM value equal 0 are marked in red. **a** Proposed anaerobic degradation of *n*-alkane by Bin9. Orange arrows indicate pathways that are associated with acetogenesis; blue arrows represent pathways that are associated with complete oxidation of *n*-alkanes coupled to sulfate reduction. **b** Proposed aerobic *n*-alkane degradation by Bin1 based on genomic information

To gain insight into the potential metabolism of microbes present in this oil reservoir, we analyzed the 44 GBs for genes and pathways associated with anaerobic degradation of hydrocarbons (see Additional file 5: Table S5). *Archaeoglobus*-like Bin9 contained the putative gene *assA*, which encodes the catalytic subunit of an alkylsuccinate synthase, an enzyme responsible for activation of hydrocarbons by fumarate addition [12] (Fig. 2a). Phylogenetic analysis of the putative *assA* gene showed a close relationship to *assA* gene in *Archaeoglobus fulgidus* (Accession number: AAB89800, see Additional file 6: Figure S1). A gene encoding alkylsuccinate synthase activating enzyme (*assD*) was also detected in Bin9 [13]. Genes for utilization of activated hydrocarbons were identified in Bin9, too. These genes include the putative CoA-synthetase/ligase (*assK*), alpha-methylacyl-CoA racemase, methylmalonyl-CoA mutase (*mcmLS*), and methylmalonyl-CoA decarboxylase (*mcd*) [14, 15]. Independent of the activation mechanism, genes involved in β-oxidation and subsequent utilization of acetyl-CoA are required for oxidation of hydrocarbons. Bin9 contained the complete β-oxidation pathway and an archaeal type Wood-Ljungdahl (WL) pathway (except the alpha and gamma subunit of formyl-MF dehydrogenase, i.e., *fwdA* and *fwdC*). Bin9 also contained the complete pathway for dissimilatory sulfate reduction, namely sulfate adenylyltransferase (*sat*), adenylylsulfate reductase (*aprAB*), and the dissimilatory sulfide reductase (*dsrABC*). Similar pathways have been identified in the genome of the isolate *Archaeoglobus fulgidus* VC-16 [13]. In addition, five copies encoding ADP-forming acetyl-CoA synthetase (*acd*) were detected in Bin9, while strain VC-16 only contains three copies [13]. Despite the presence of *ehbQ* (energy-converting hydrogenase B, subunit Q) and *frcB* (F_420_-reducing hydrogenase, subunit beta), genes encoding for complete enzymes which are necessary for H_2_ or formate production, such as [FeFe] hydrogenase, [NiFe] hydrogenase or formate dehydrogenase, were absent in Bin9 [16].

In *Methanosaeta*-like Bin39, besides acetyl-coenzyme A synthase (*acs*) and acetyl-coenzyme A decarbonylase/synthase complex (*codhABCDE*) that are involved in acetoclastic methanogenesis, we also found genes involved in reduction of CO_2_ to methane, namely formyl methanofuran dehydrogenase (*fwdABCDEFG*), and also formyl methanofuran–H_4_MPT formyl-transferase (*ftr*), methenyl-H_4_MPT cyclohydrolase (*mch*), F_420_-dependent methylene-H_4_MPT dehydrogenase (*mtd*), F_420_-dependent methylene H_4_MPT reductase (*mer*), tetrahydromethanopterin (H_4_MPT)-*S*-methyltransferase (*mtd*), methyl-CoM reductase (*mcrABCDG*) and heterodisulfide reductase (*hdrABCDG*) (see Additional file 7: Figure S2). No formate dehydrogenase or hydrogenase was found in this bin.

Apart from genes associated to anaerobic hydrocarbon degradation, we also identified genes for aerobic hydrocarbon degradation from these wells. The integral-membrane alkane hydroxylase (*alkB*), which catalyze the terminal oxidation of medium/long chain (> C_6_) alkanes in the presence of oxygen [17], a rubredoxin, an electron transfer protein required by alkane hydroxylase [18], and a NAD(P)H-dependent rubredoxin reductase (*rubB*), were identified in the *Acinetobacter*-like Bin1 (Fig. 2b). In addition, a gene encoding for a Baeyer-Villiger monooxygenase (*almA*), which is involved in subterminal oxidation of long-chain alkanes (C_20_-C_32_) [19], was identified in Bin1. Further analysis revealed that all genes necessary for processing of activated alkanes, such as alcohol dehydrogenase (*adh*), aldehyde dehydrogenase (*aldh*), and genes for β-oxidation, were also present in Bin1. *alkB* was also identified in *Pseudomonas*-like Bin19. However, likely due to low genome completeness of Bin19 (30.3%), only *adh* and *aldh* were additionally detected in this GB.

### Transcriptional activity of community members

**Fig. 3:**
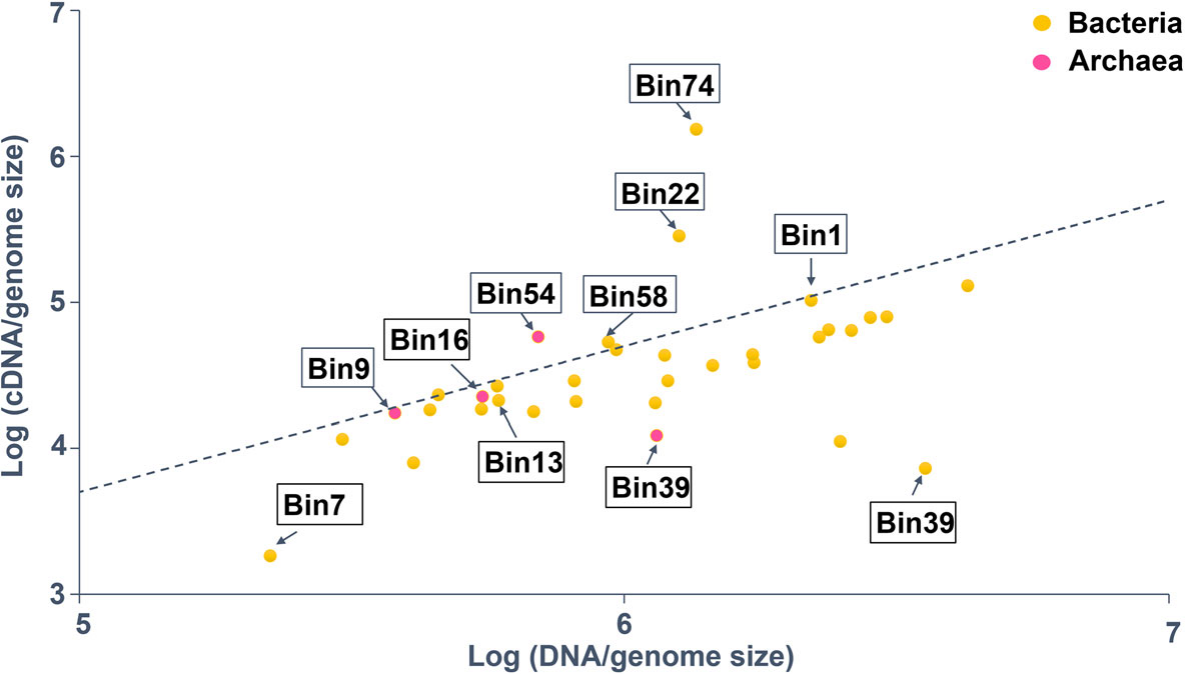
DNA and cDNA mapping rate of GBs for sample W15. The GBs above the dashed line showed cDNA/DNA > 0.5

Given the fact that microbial communities in three samples are similar (Fig. 1), RNA extracted from sample W15 was chosen as a representative to study microbial metabolic activity in situ by using a metatranscriptomics approach [20] The cDNA sequencing generated ~ 20 million reads, and the sequence coverage estimated by Nonpareil [10] was very high (97%) for W15. Metatranscriptomic reads were mapped to CDSs from previously assembled GBs, and the mapping rates of metatranscriptomic reads were normalized by the mapping rates of metagenomics reads from the same sample (W15) (Fig. 3). This analysis identified the most active microorganisms in situ. *Pseudomonas*-like Bin74 had the highest cDNA/DNA ratio (11.3), followed by Clostridia unclassified Bin22, Euryarcheota unclassified Bin54, Bacteria unclassified Bin58, Alphaproteobacteria unclassified Bin72, and Betaproteobacteria unclassified Bin71 (≥ 0.5). *Archaeoglobus*-like Bin9, Bin16 and *Methanosaeta-*like Bin39 exhibited a cDNA/DNA ratio of 0.45, 0.1, and 0.41.

Metatranscriptomic sequence reads were mapped to co-assembled contigs to obtain genome-specific gene expression (see Additional file 8: Table S6). Genes with FPKM value > 10, which ranked in the top 25% of all genes, were defined as highly transcribed (see Additional file 9: Figure S3). According to transcription analysis, the *Archaeoglobus*-like Bin9 transcribed the putative genes *assA* and *assD* at high level (FPKM > 10) (Fig. 2a). Genes involved in downstream pathways of alkane activation were also highly transcribed with FPKM values ranging from 30 to 70 in this genome. While genes for β-oxidation were highly transcribed (FPKM > 30), methenyl-H_4_MPT cyclohydrolase gene (*mch*) of the WL pathway was transcribed at low level (FPKM < 10). Genes involved in sulfate reduction (*dsrA* and *arpB*) were transcribed at a very low level (FPKM < 5), and transcription of *dsrC*, the key gene for dissimilatory sulfate reduction [21], was below detection limit (FPKM = 0). By contrast, genes encoding the ADP-forming acetyl-CoA synthetase (*acd*) were highly expressed in Bin9 (total FPKM > 40).

Nearly all genes involved in acetoclastic methanogenesis and CO_2_-reductive methanogenesis, except for methyl-CoM reductase, subunit gamma (*mcrC*) and subunit B and F of tetrahydromethanopterin methyltransferase (*mtrB*, *mtrF*), were transcribed to different degree (see Additional file 8: Table S6).

Concurrently to anoxic alkane degradation, genes involved in oxic degradation of hydrocarbons were also highly transcribed (see Additional file 8: Table S6). Genes involved in aerobic alkane degradation from *Acinetobacter*-like Bin1 were highly expressed with FPKM value of *almA* = 62, *alkB* = 33, *rubA* = 28 and *rubB* = 103 (Fig. 2b, also see Additional file 8: Table S6). Genes involved in metabolizing activated alkanes, such as alcohol and aldehyde dehydrogenase and genes associated with β-oxidation, were also highly transcribed in this GB with FPKM values > 80. In comparison, the *alkB* gene of *Pseudomona*s-like Bin19 was transcribed at a significantly lower level (FPKM = 5.6), suggesting that *Acinetobacter*-like Bin1 contributes more to aerobic alkane oxidation in well W15.

### Amino acid and vitamin auxotrophies

**Fig. 4:**
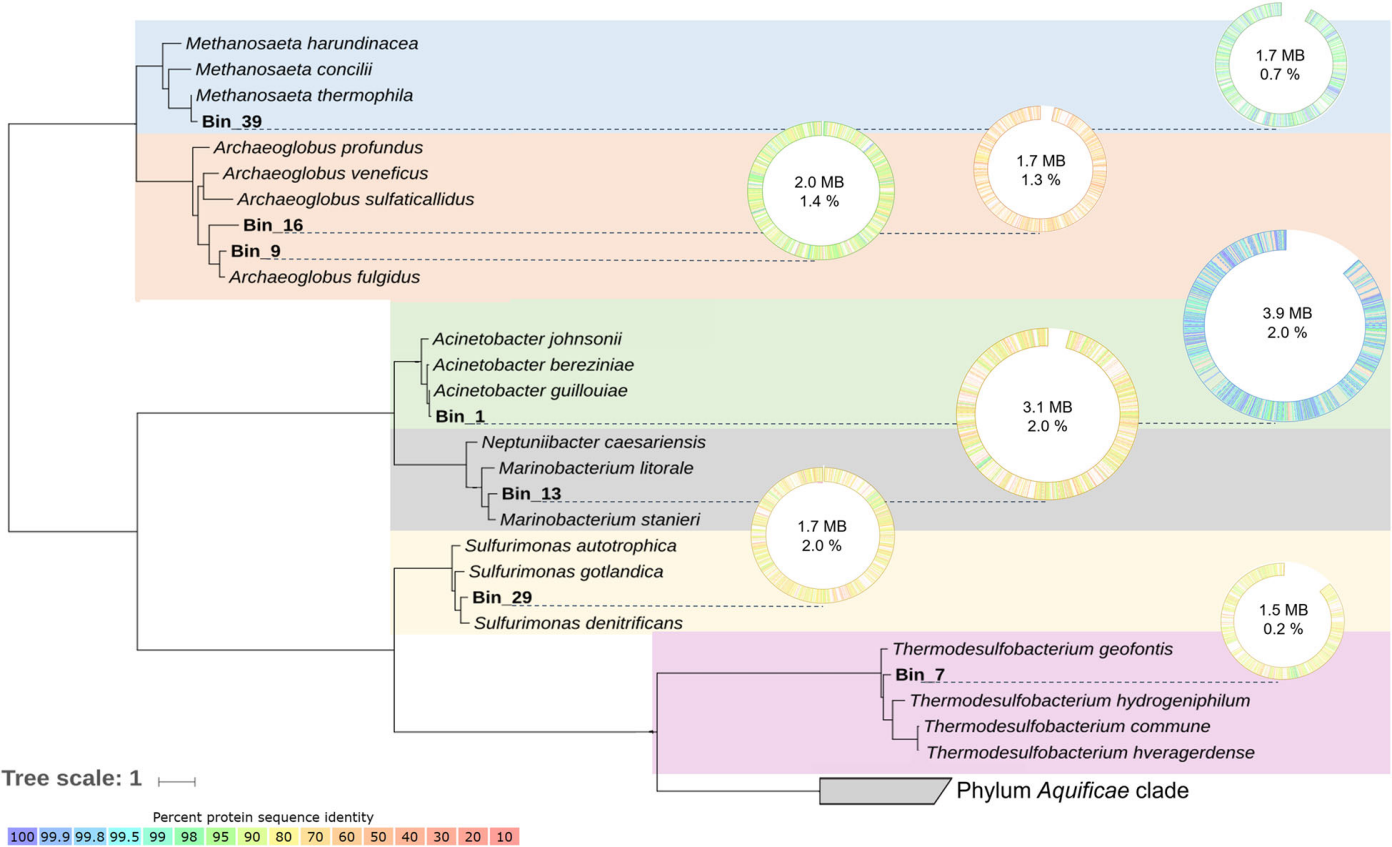
Phylogenetic tree of high-quality GBs. Circles represent each genome. The completeness of genomes is shown as the radian, and similarities of amino acid sequences to reference genomes are indicated by color. Genome size and estimated contamination rate are provided inside each circle

**Fig. 5:**
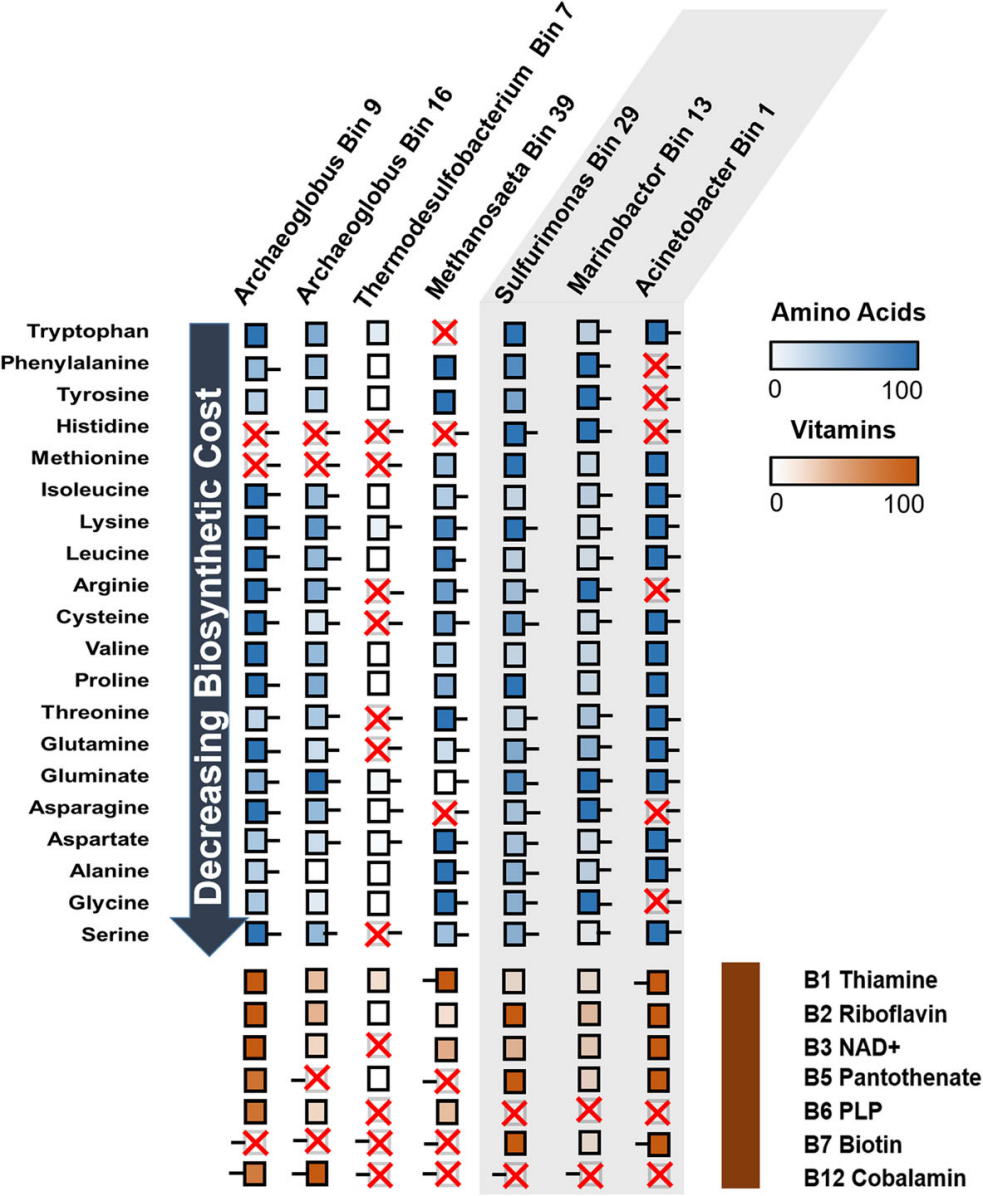
Specific amino acid auxotrophies and vitamin auxotrophies present in high-quality GBs. The auxotrophy of specific compound was confirmed if more than one gene was absent in the synthetic pathway, or overall transcriptional level of pathway was low compared to genomes with complete pathway in which only one gene was absent. Aerobic species are covered in gray. Amino acids are in depicted in blue and vitamins in brown. Amino acids have been ranked according to biosynthetic cost (arrow). A colored square denotes that a species can synthesize an amino acid. The intensity of each color (based on the scale) represents the relative expression of the synthesis pathways, with darker/more intense color indicating higher expression. Auxotrophies are marked by a red cross. A black line next to the box represents the presence of a transporter. A transport system is not required for vitamins B2 and B3

Auxotrophies and exchange of amino acids and vitamins have been shown to reinforce interactions in a syntrophic alkane-oxidizing community in the laboratory [20, 22]. We therefore analyzed the seven nearly complete GBs (> 85% complete), i.e., *Archaeoglobus* Bin9 and Bin16, *Thermodesulfobacterium* Bin7, *Methanosaeta* Bin39, *Sulfurimonas* Bin29, *Marinobacter* Bin13, and *Acinetobacter* Bin1 from the oil reservoir for amino acid and vitamin auxotrophies (Fig. 4). In specific, the four most abundant anaerobic microorganisms had the capacity to synthesize all essential amino acids but histidine, and all vitamins but B7. A similar result was observed for the three most complete GBs of aerobic community members. While the three aerobic microbes where not able to synthesize vitamins B6 and B12, amino acid auxotrophies were much less abundant in these organisms, with *Sulfurimonas* and *Marinobacter* being able to synthesize all amino acids (Fig. 5).

## Discussion

Microbial utilization of hydrocarbons from crude oil has long been known to occur under both oxic and anoxic conditions. While pristine oil reservoirs were considered mainly anoxic, insertion of injection water during secondary oil recovery introduces molecular oxygen to these environments, generating suitable conditions for both aerobic and anaerobic microbial communities to thrive.

Dominant taxa identified by shotgun metagenomics in this study were also the most abundant microorganisms detected by an amplicon metagenomics approach [11]. Microorganisms associated to taxa generally consisting of obligate aerobic bacteria, such as *Acinetobacter* and *Marinobacter*, were abundant in all three wells from the Jiangsu oil reservoir. These aerobic bacteria are believed to participate in the degradation of hydrocarbons in the oil reservoir using molecular oxygen [6, 23]. Facultative anaerobes, *Pseudomonas* species have been frequently detected in oil reservoirs [24, 25], and some members of them are capable of degrading aromatic hydrocarbons or alkanes [26, 27]. Although anaerobic archaea, such as *Archaeoglobus*, as well as *Methanosaeta* were present at lower abundance than aerobic bacteria, they were found with relatively high transcript level in W15 sample, suggesting their important roles in oil reservoir. *A. fulgidus* strain VC-16, a representative of the genus *Archaeoglobus*, isolated from marine hydrothermal systems can degrade *n*-alkanes (C_10_-C_21_) with thiosulfate or sulfate as terminal electron acceptor [28]. The mapping rate of metatranscriptomic reads implies concurrent activity of aerobic microorganisms, such as *Pseudomonas* and *Acinetobacter*, and anaerobic microorganisms such as *Archaeoglobus* and *Methanosaeta* in this well.

### Acetogenic fermentation of alkanes

While the exact mechanism of hydrocarbon activation in *A. fulgidus* VC-16 or the *Archaeoglobus*-like Bin9 is still unclear at the time, it is assumed that a glycyl radical enzyme (AssA) is responsible for the activation in *A. fulgidus* VC-16 [28]. A gene sequence annotated as *assA* has been identified in Bin9. While Bin9 is capable of sulfate respiration, the presence of *acd* and genes for *β*-oxidation in Bin9 suggests that this microorganism can ferment organic compounds, like fatty acids or *n*-alkanes, when electron acceptors are depleted [29]. Moreover, gene expression indicates that most cells are fermenting under in situ conditions using the WL pathway (Fig. 2a). In this process, acetate formation from acetyl-CoA couples with reductive acetogenesis from CO_2_, and energy was conserved using Acd during hydrocarbon degradation [29]. According to thermodynamic calculation as proposed by Dolfing et al. [30], pH and acetate concentration found in the Jiangsu oil reservoir would also support incomplete oxidation of alkanes to acetate in these wells (see Additional file 10: Table S7 and Additional file 11: Figure S4) [30, 31]. Low levels of sulfate and absence of sulfide in well W15, as well as lack of expression of *dsrC* further support a fermentative rather than respiratory life style of the alkane-degrading *Archaeoglobus*-like Bin9 (see Additional file 12: Table S8 and full details are in Additional file 13: Text S1) in the reservoir. Based on genomic and transcriptomic evidence, we speculated that *Archaeoglobus*-like Bin9 ferments hydrocarbons to acetate in situ, which is consumed by other microorganisms.

### Potential methanogenic activity via direct interspecies electron transfer

The presence of *Methanosaeta* in the samples hints at methanogenic degradation of hydrocarbons [20]. Although methane was not measured in the samples, methanogenic enrichment cultures could be obtained from these samples. The expression analysis indicated active acetoclastic methanogenesis and CO_2_-reductive methanogenesis in well W15. The absence of formate dehydrogenase or hydrogenase in this bin was consistent with studies on other *Methanosaeta* species, suggesting direct interspecies electron transfer (DIET) when CO2 serves as electron acceptor for these methanogens [32, 33]. However, one should bear in mind that Bin39 represents an incomplete genome sequence and while formate dehydrogenase and hydrogenase are absent in other *Methanosaeta* genomes, these genes could be present in Bin39. The high expression of *fwdG* (FPKM > 80) also suggested CO_2_-reduction to occur via DIET in addition to acetoclastic methanogenesis, as describe before [20, 32, 34].

### Complete oxidation of alkanes

The detection of alkane hydroxylase, together with genes encoding for rubredoxin and rubredoxin reductase in *Acinetobacter*-like Bin1, indicate the ability of this microorganism to oxidize alkanes aerobically. The co-occurrence of different types of alkane hydroxylases (*alkB* and *almA*) implied that Bin1 could potentially utilize different strategies for degrading alkanes with variable chain length [17, 19]. A putative pathway for complete oxidation of *n*-alkanes by Bin1 was reconstructed (Fig. 2b) [35]. The high levels of transcription of genes associated to hydrocarbon degradation suggest that *Acinetobacter* actively participates in the oxic degradation of hydrocarbons in the oil reservoir.

### Amino acid and vitamin auxotrophies

None of the seven GBs had all genes required for synthesizing all amino acids and vitamins, hinting at complex interdependencies among microorganisms in this oil reservoir (Fig. 5), similar to what has been described for communities of both long- and short-chain hydrocarbon-degrading communities from enrichment cultures [20, 22]. The absence of microbial members that synthesize specific vitamins and amino acids in the aerobic and anaerobic communities suggested that additional partners are needed for these communities to thrive. According to the result, intertwined dependencies based on amino acid auxotrophy thus seem to be more prevalent in the anoxic community. Interestingly, amino acids auxotrophies in the aerobic microorganisms were not as dominant as in the anaerobic microorganisms. Reduced energy yields for microorganisms growing with hydrocarbons under anoxic conditions compared to oxic conditions might be responsible for dominant amino acid auxotrophies observed here and in a previous study based on laboratory communities [20, 22]. Individual aerobic microbes from this oil reservoir are capable for complete hydrocarbon oxidation, diminishing their requirement for metabolic interaction. However, all amino acids and vitamins could be synthesized when both aerobes and anaerobes were taken as a unit.

The prevalence of amino acid and vitamin auxotrophies within the key members of the anaerobic community in the Jiangsu oil reservoir hint at a highly intertwined network of interactions. A similar network has been described in a methanogenic alkane- and fatty acid-degrading communities where very few members were capable of histidine synthesis [20]. While histidine auxotrophies were most prevalent among community members investigated here, auxotrophies of *Methanosaeta*-like Bin39 were different to what was previously reported for a *Methanosaeta* strain in syntrophic communities propagated in the laboratory [20], indicating that interactions are dependent on individual strains and can vary based on community composition. Further investigations of amino acid auxotrophies are needed to reveal if this is a hallmark of syntrophic communities degrading hydrocarbons anaerobically. While this study demonstrated the concurrent activity of different communities, it is currently unknown how these communities are organized in time and space. Thus, studies are needed that account for the spatial organization of members of the aerobic and anaerobic communities, e.g., by SIP or NanoSIMS.

## Conclusions

While aerobic and anaerobic microorganisms have simultaneously been identified from oil reservoirs undergoing secondary recovery, it has often been difficult to delineate what role these organisms are playing for the microbial ecology of the oil reservoir. The main reason is that injection waters carry large number of microbes that can contribute significantly to abundance profiles from these reservoirs but are not actively participating in the degradation of oil hydrocarbons. Here, we elucidated the metabolic potential of individual members of thermophilic microbial communities from an oil reservoir in Jiangsu, China, by metagenomics and determined their role in hydrocarbon metabolism in situ by metatranscriptomics. We identified key members of the aerobic and anaerobic community performing oxic or anoxic hydrocarbon degradation and determined that a large fraction of microorganisms detected by omics approaches were mostly inactive, likely originating from the injection water. Along with potential metabolic exchanges, we identified prevalent auxotrophies in members of this thermophilic community, hinting at a complex network of interactions. While both aerobic and anaerobic communities are active in the oil reservoir at the same time, sampling at high spatial resolution is required to delineate potential interactions and exchanges between these two communities.

## Methods

### Samples of production water

Production water samples for DNA and RNA isolation were collected on March, 19, 2015, from three different wells, i.e., W2-71, W9-18 and W15-5, in the Jiangsu Oil Reservoir (Jiangsu, China). Forty liters of production water was collected for each sample for DNA extraction, and another 40 L of production water for RNA extraction was stabilized using a 10% (vol/vol) stop solution (95% ethanol, 5% TRIzol (Life Technology)) [34]. All samples were kept on ice and transported to the laboratory within 4 h and immediately centrifuged at 12,000×*g* at 4 °C for 10 min upon arrival in the lab. After removal of supernatant, pellets were kept at − 80 °C and processed within 2 weeks.

### Metagenomic and metatranscriptomic sequencing

DNA and RNA were extracted from pellets using the PowerMicrobiome™ RNA Isolation Kit (MO BIO). To enrich messenger RNA (mRNA), ribosomal RNA was depleted from total RNA using Ribo-Zero rRNA Removal Kit (Bacteria) (Illumina®) according to the manufacturer’s instructions. Double-strand cDNA was synthesized as described before [34]. Sequencing libraries for both metagenomic and metatranscriptomic were prepared using the Nextera XT DNA Library Prep Kit (Illumina®). The quality of the libraries was checked using a Bioanalyzer High Sensitivity Chip (Agilent), and the libraries were quantified by Qubit dsDNA HS Assay. The metagenomics libraries were sequenced on an Illumina Miseq using 150-cycle kit (2 × 75 pb paired-end). The metatranscriptomic libraries were sequenced on the same platform using a 50-cycle kit.

### Taxonomic assessment of unassembled metagenomic reads

Filtered metagenomic reads were used for taxonomic assessment by screening for SSU rRNAs with Metaxa2 v2.1.3 [36] and for phylogenetic marker genes using MetaPhlAn2 v2.7.0 [37].

### Metagenome assembly and analysis

The metagenomic raw reads were examined using FastQC tool (http://www.bioinformatics.babraham.ac.uk/projects/fastqc/), and low-quality sequences were trimmed using PRINSEQ v0.20.4 (parameters: ‘trim_tail_right’, 5; trim_tail_left’, 5; entropy threshold, 50; minimum read length of 70 base; maximum replication, 6; maximum ‘n’s, 0) [38]. The level of coverage of each metagenomic dataset was estimated using Nonpareil with default parameters [10]. The trimmed reads were independently assembled de novo using SPAdes v3.7.0 with the following parameters ‘--meta -k 33, 45, 61, 71’. The contigs of each metagenome were annotated using MG-RAST online server [39]. To generate a reference dataset for further additional analysis, trimmed reads from the three metagenomes were combined and co-assembled using SPAdes.

Ribosomal genes were identified using two complementary methods. In summary, (1) the dominant species (based on reconstructed near-full-length 16S rRNA gene) of each metagenome were identified using EMIRGE with default parameters [40] and (2) rRNA_hmm [41] was used to search assembled contigs containing 16S rRNA sequences. 16S rRNA sequences > 240 bp identified by EMIRGE and rRNA_hmm were combined for further analysis. Redundant 16S rRNA genes were manually evaluated by blasting [42] each EMIRGE-reconstructed sequence to rRNA_hmm-found sequences. If the alignment showed 100% of identity, the rRNA_hmm sequence was discarded. 16S rRNAs chimeric sequences were identified and deleted using DECIPHER [43]. In order to generate representative OTUs, non-chimeric sequences were clustered using CD-HIT v4.6 on 97% of similarity [44]. Ribosomal Database Project (RDP) were used to assign the OTUs to their corresponding taxonomy [45].

### Screening for functional genes

Metagenomic reads from three samples were combined to get better assembly and greater coverage of the genomic bins [46] (full details in Additional file 14: Text S2). Co-assembled contigs were examined for the presence of genes associated with oxic and anoxic hydrocarbon-degrading pathways [47, 48]. Hidden Markov Models (HMMs) for translated sequences were constructed *in-house* using HMMER (v3.1b1) [49]. To construct HMMs, amino-acid sequences for hydrocarbon-degrading pathways proteins were obtained from GenBank and aligned using MAFFT. Multiple alignments were manually curated using AliViewv 1.18 [50]. Curated alignments were used as an input to HMMER to create HMMs. The HMMs were used to search for hydrocarbon-degrading proteins among the translated genes obtained from the co-assembled metagenome using Prodigal v2.6 [51]. If the number of sequences were not enough to build a HMM, individual protein sequences were searched against the metagenome translated genes using BLASTP with *E*-value < e^−10^ [42]. All hits were manually curated using NCBI NR database and false positives were discarded.

### Differential binning of metagenomic reads

Co-assembled contigs were used for differential binning [52]. In brief, individual trimmed reads were mapped back to the contigs file, and the resulting bam files were used as input into GroopM with default parameters (v0.3.4) [52]. De-contamination of retrieved genome bins (GBs) was carried out in ProDeGe [53]. Bonafide GBs were uploaded to RAST [54] for annotation. All GBs were checked for completeness and contamination using checkM v1.0.5 [55].

### Genome analysis

PhyloPhlAn v0.99 [56] was used to reconstruct the phylogenetic tree of all GBs based on the protein prediction results from Prodigal v2.6 [51]. Additionally, we used BLAST [42] to compare the 16S rRNA gene annotated in each bin. Amino acid synthesis pathway, vitamin synthesis pathways, and vitamins transport system in near-complete GBs were curated manually using primary literature and the KEGG database [57] as previously described [20]. Metatranscriptomic dataset of W15 was used in addition to help curate amino acid and vitamin synthesis pathways**.** For biosynthetic pathways in which only one gene was missing, we used metatranscriptomic information to evaluate expression levels of the pathway. If the overall transcription level was similar or higher than in GBs that harbor a complete pathway, this pathway was considered complete.

### Metatranscriptome analysis

Metatranscriptomic raw reads were trimmed by quality using Prinseq (parameters were identical to the metagenome analysis), and trimmed reads were mapped to GB’s coding sequences (CDS) using Bowtie2 [58] with default settings. Transcription level of each recovered GB was evaluated by computing the cDNA/DNA abundance ratio. Transcription level of individual genes in each GB was determined by mapping metatranscriptomic reads to co-assembled contigs using Bowtie2 [58] with default parameters. eXpress v1.5.1 [59] was used to calculate FPKM (Fragments Per Kilobase of transcript per Million) values, and the genes whose FPKM values rank in top 25th percentile were defined as actively expressed.

## Additional files

Additional file 1: Table S1. Summary of assembled contigs. (DOCX 14 kb)

Additional file 2: Table S2. Estimate of metagenomic sequencing coverage. (DOCX 21 kb)

Additional file 3: Table S3. Taxonomic classification of assembled 16S rRNA gene sequences from metagenomes of W2, W9 and W15. (DOCX 31 kb)

Additional file 4: Table S4. Information of genome bins (separate file). The completeness, contamination and strain heterogeneity of GBs were evaluated by CheckM. The columns for taxonomy were splited into three, within which the ‘ProDeGe_Tax’ column recorded the information from ProDeGe server based on BlastP search, the ‘Rast_Closet_Spp.’ column recorded information from RAST server using its ‘View closest neighbors’ function and the ‘Phylophlan_Assignment’ column recorded the lineage output using Phylophlan. (XLSX 18 kb)

Additional file 5: Table S5. Information of GBs studied in context. (DOCX 17 kb)

Additional file 6: Figure S1. Phylogenetic tree of amino acid sequences of *assA* genes. (PNG 287 kb)

Additional file 7: Figure S2. Proposed Syntrophic model for (a) *Methanosaeta* and (b) *Archaeoglobus*. The expression level of each gene is represented by bars (pink and blue), with one bar representing FPKM value of 10. Genes for which not transcripts could be mapped (FPKM value = 0) are marked in red. (TIFF 1778 kb)

Additional file 8: Table S6. Annotation and FPKM value of hydrocarbon degradation and methanogenesis related genes in GBs (separate file). Annotation of studied GBs was done by a combined effort of RAST server and KEGG database. (XLSX 429 kb)

Additional file 9: Figure S3. Cumulative distribution plot of genes under certain FPKM value. Scatter plot of accumulated number of genes in co-assembled scaffolds under certain FPKM value based on the metatranscriptome W15. Genes with FPKM value less than 10 accounted for 75% of total genes, therefore genes with FPKM above 10 were defined as actively expressed genes. (TIFF 396 kb)

Additional file 10: Table S7. Thermodynamic constraint of acetate concentration on oxidation of hexadecane to acetate. (DOCX 15 kb)

Additional file 11: Figure S4. Effect of pH on the range of acetate concentrations when n-alkanes (i.e. hexadecane as representative for calculation) are oxidized to acetate as sole fermentation product and coupled to acetoclastic methanogenesis. The green line represents the oxidation of hexadecane to acetate; red line represents the conversion of acetate to CH_4_. Blue dots represent acetate concentration and pH values present in wells W2, W9 and W15. The arrows indicate conditions under which the process becomes exergonic. The highlighted zone (light blue) indicates a window of opportunity where oxidization of hexadecane to acetate coupled to acetoclastic methanogenesis is thermodynamically favorable. The figure was modified from [30]. (TIFF 682 kb)

Additional file 12: Table S8. Geochemical characterization of production water from three wells from the Jiangsu oil field, China. (* ND, not detected.) (DOCX 15 kb)

Additional file 13: Text S1. Supplementary results and discussion. (DOCX 17 kb)

Additional file 14: Text S2. Supplementary materials and methods. (DOCX 29 kb)
