# Supplementary material for: Metabolic capability and in situ activity of microorganisms in an oil reservoir
Source: Microbiome. 2018 Jan 5;6:5. doi: 10.1186/s40168-017-0392-1 (PMC5756336; doi:10.1186/s40168-017-0392-1)
Supplement: Supplementary file 1 — Summary of assembled contigs. (DOCX 14 kb) [file 40168_2017_392_MOESM1_ESM.docx]

**Table S1 | Summary of assembled contigs.**

| Contigs | W2 | W9 | W15 | Co-assembly |
| --- | --- | --- | --- | --- |
| Number of reads | 9,187,244 | 10,868,754 | 7,727,828 | 27,783,826 |
| Number of contigs | 42897 | 23709 | 51189 | 62850 |
| Largest contig (bp) | 532445 | 135232 | 144480 | 531922 |
| Total length (bp) | 37562366 | 23532642 | 50052376 | 70211042 |
| N50 (bp) | 1538 | 2618 | 733 | 4717 |
| GC (%) | 55.6 | 43.43 | 53.82 | 52.73 |
