# Supplementary material for: Metabolic capability and in situ activity of microorganisms in an oil reservoir
Source: Microbiome. 2018 Jan 5;6:5. doi: 10.1186/s40168-017-0392-1 (PMC5756336; doi:10.1186/s40168-017-0392-1)
Supplement: Supplementary file 2 — Estimate of metagenomic sequencing coverage. (DOCX 21 kb) [file 40168_2017_392_MOESM2_ESM.docx]

**Table S2 | Estimate of metagenomic sequencing coverage.**

| Sample | W2 | | W9 | | W15 | |
| --- | --- | --- | --- | --- | --- | --- |
| Read | R1 | R2 | R1 | R2 | R1 | R2 |
| Coverage (%) | 80.64 | 80.43 | 94.03 | 94.08 | 88.53 | 86.62 |
