# Supplementary material for: Metabolic capability and in situ activity of microorganisms in an oil reservoir
Source: Microbiome. 2018 Jan 5;6:5. doi: 10.1186/s40168-017-0392-1 (PMC5756336; doi:10.1186/s40168-017-0392-1)
Supplement: Supplementary file 3 — Taxonomic classification of assembled 16S rRNA gene sequences from metagenomes of W2, W9 and W15. (DOCX 31 kb) [file 40168_2017_392_MOESM3_ESM.docx]

**Table S3 | Taxonomic classification of 16S rRNA gene sequences in metagenomes of W2, W9 and W15.**

| OTU No. | Taxonomic affiliation (order level) | W2 | W9 | W15 |
| --- | --- | --- | --- | --- |
| 1 | Thermodesulfobacteriales | 0 | 6 | 1 |
| 2 | Clostridiales | 1 | 0 | 1 |
| 3 | Thermoanaerobacterales | 0 | 1 | 1 |
| 4 | Pseudomonadales | 7 | 8 | 3 |
| 5 | Alteromonadales | 5 | 7 | 1 |
| 6 | Burkholderiales | 0 | 1 | 2 |
| 7 | Campylobacterales | 8 | 7 | 4 |
| 8 | Rhodocyclales | 1 | 0 | 0 |
| 9 | Methanosarcinales | 0 | 5 | 1 |
| 10 | Archaeoglobales | 1 | 1 | 1 |
| 11 | Unclassified | 16 | 8 | 4 |
