# Supplementary material for: Metabolic capability and in situ activity of microorganisms in an oil reservoir
Source: Microbiome. 2018 Jan 5;6:5. doi: 10.1186/s40168-017-0392-1 (PMC5756336; doi:10.1186/s40168-017-0392-1)
Supplement: Supplementary file 5 — Information of GBs studied in context. (DOCX 17 kb) [file 40168_2017_392_MOESM5_ESM.docx]

**Table S5 | Information of GBs studied in context**

| **GB** | **Size (Mb)** | **Completeness* (%)** | **Association** | **DNA %/M in W2** | **DNA %/M in W9** | **DNA %/M in W15** | **cDNA %/M in W15** | **Genes involved in hydrocarbon degradation** | **Genes mentioned in the main text** |
| --- | --- | --- | --- | --- | --- | --- | --- | --- | --- |
|  |  |  |  |  |  |  |  |  |  |
| Bin29 | 1.9 | 99.6 | *Sulfurimonas* | 4.7 | 0.8 | 4.7 | 0.1 |  |  |
| Bin9 | 2 | 99.4 | *Archaeoglobus* | 0.2 | 2.9 | 0.5 | 0.1 | *assA*; *assD* | *dsrABC*; *aprAB*; *Sat*; *mcmSL*; *mcee*; *mcd*; *sucC*; *sucD*; *sdhA*; *acadm;* genes involved in *β*-oxidation; genes involved in Wood-Ljungdahl pathway |
| Bin16 | 1.7 | 96.5 | *Archaeoglobus* | 0.2 | 0.6 | 1.5 | 0.1 |  |  |
| Bin13 | 3.1 | 96.1 | *Marinobacter* | 1.8 | 0.2 | 0.8 | 0.1 |  |  |
| Bin39 | 1.7 | 92.3 | *Methanosaeta* | 0 | 0.7 | 0.7 | 0.1 |  | genes involved in methanogenesis |
| Bin1 | 3.9 | 87.3 | *Acinetobacter* | 0.7 | 5.7 | 2.9 | 0.3 | *almA*; *alkB* | *rubB*; *adh*; *aldh;* genes involved in *β-*oxidation |
| Bin7 | 1.5 | 86.3 | *Thermodesulfobacterium* | 0.1 | 5.5 | 0.1 | 0 |  |  |
| Bin19 | 1.2 | 30.3 | *Pseudomonas* | 0.8 | 0.1 | 1 | 0.1 | *alkB* |  |
