# Supplementary figures and images for: Metabolic capability and in situ activity of microorganisms in an oil reservoir
Source: Microbiome. 2018 Jan 5;6:5. doi: 10.1186/s40168-017-0392-1 (PMC5756336; doi:10.1186/s40168-017-0392-1)

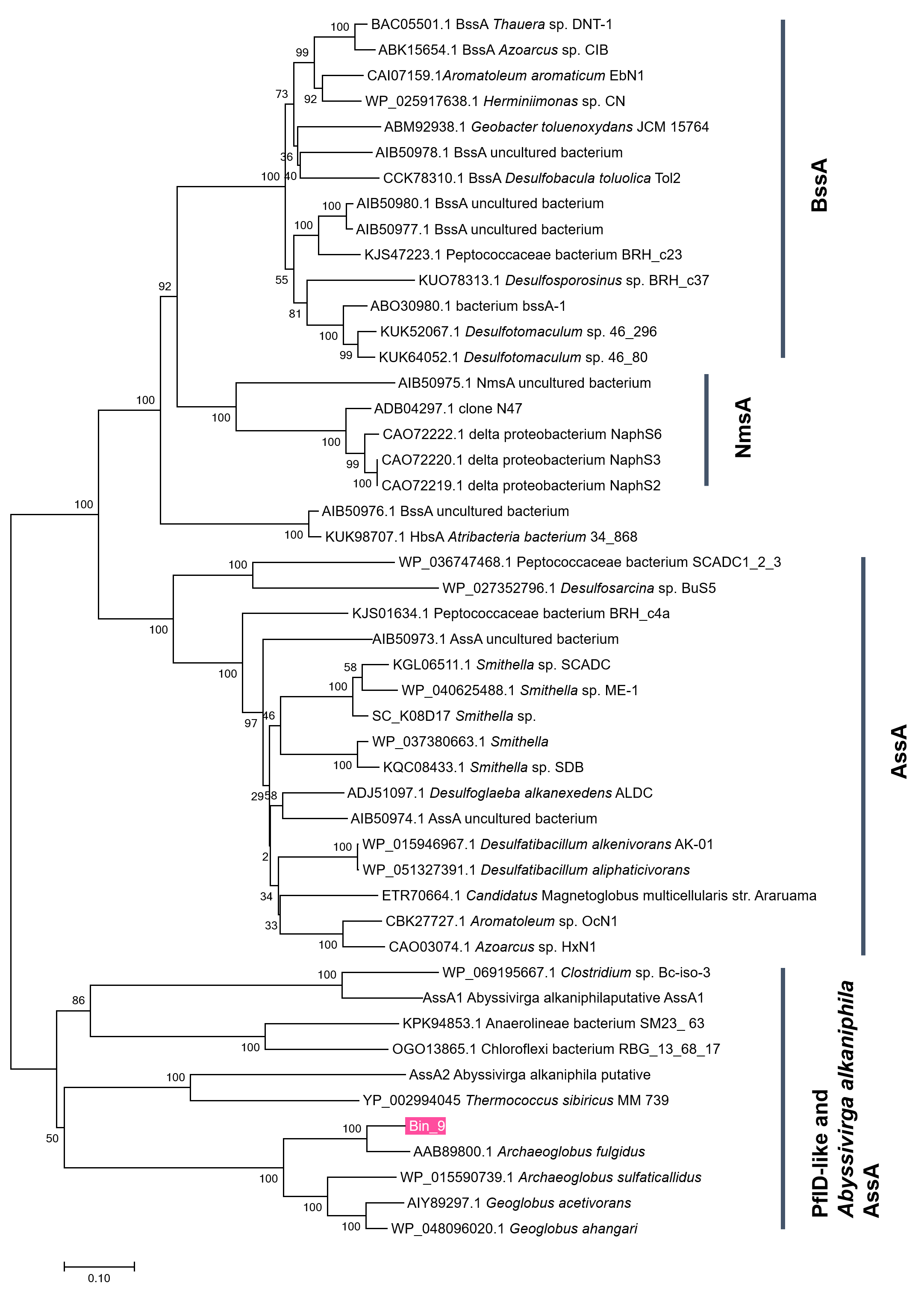

Supplement: Supplementary file 6 — Phylogenetic tree of amino acid sequences of assA genes. (PNG 287 kb) [file 40168_2017_392_MOESM6_ESM.png]

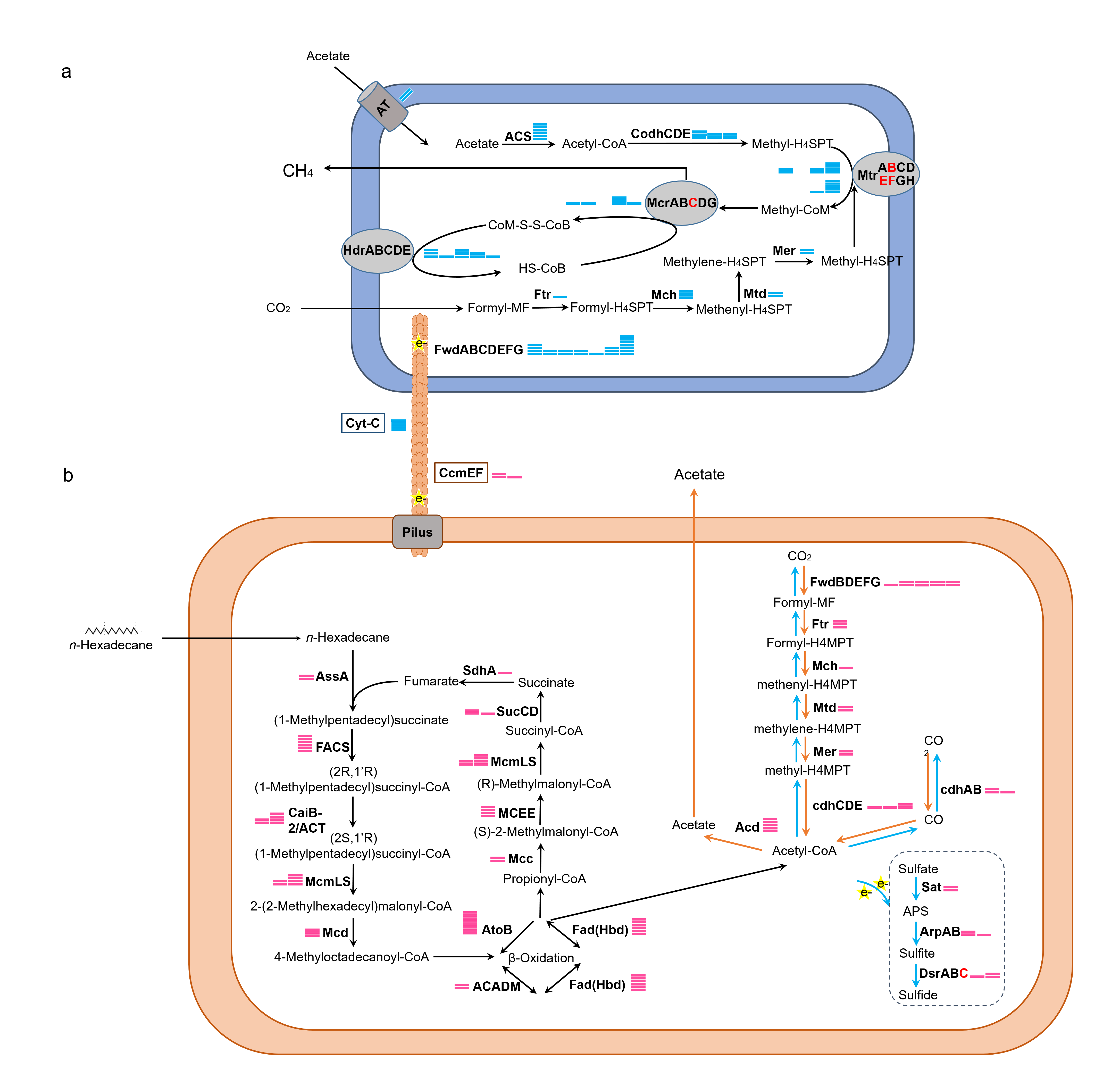

Supplement: Supplementary file 7 — Proposed Syntrophic model for (a) Methanosaeta and (b) Archaeoglobus. The expression level of each gene is represented by bars (pink and blue), with one bar representing FPKM value of 10. Genes for which not transcripts could be mapped (FPKM value = 0) are marked in red. (TIFF 1778 kb) [file 40168_2017_392_MOESM7_ESM.tif]

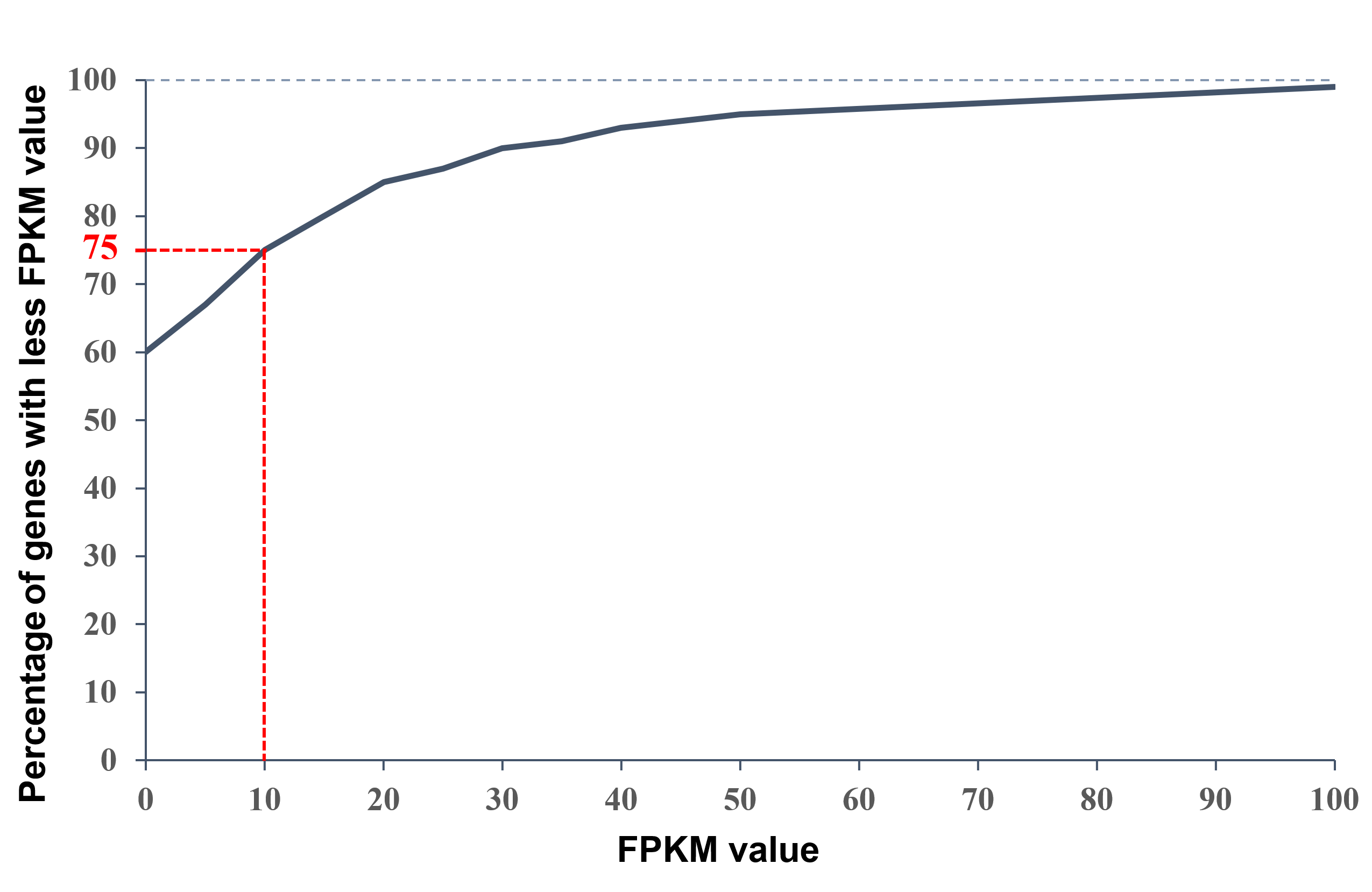

Supplement: Supplementary file 9 — Cumulative distribution plot of genes under certain FPKM value. Scatter plot of accumulated number of genes in co-assembled scaffolds under certain FPKM value based on the metatranscriptome W15. Genes with FPKM value less than 10 accounted for 75% of total genes, therefore genes with FPKM above 10 were defined as actively expressed genes. (TIFF 396 kb) [file 40168_2017_392_MOESM9_ESM.tif]

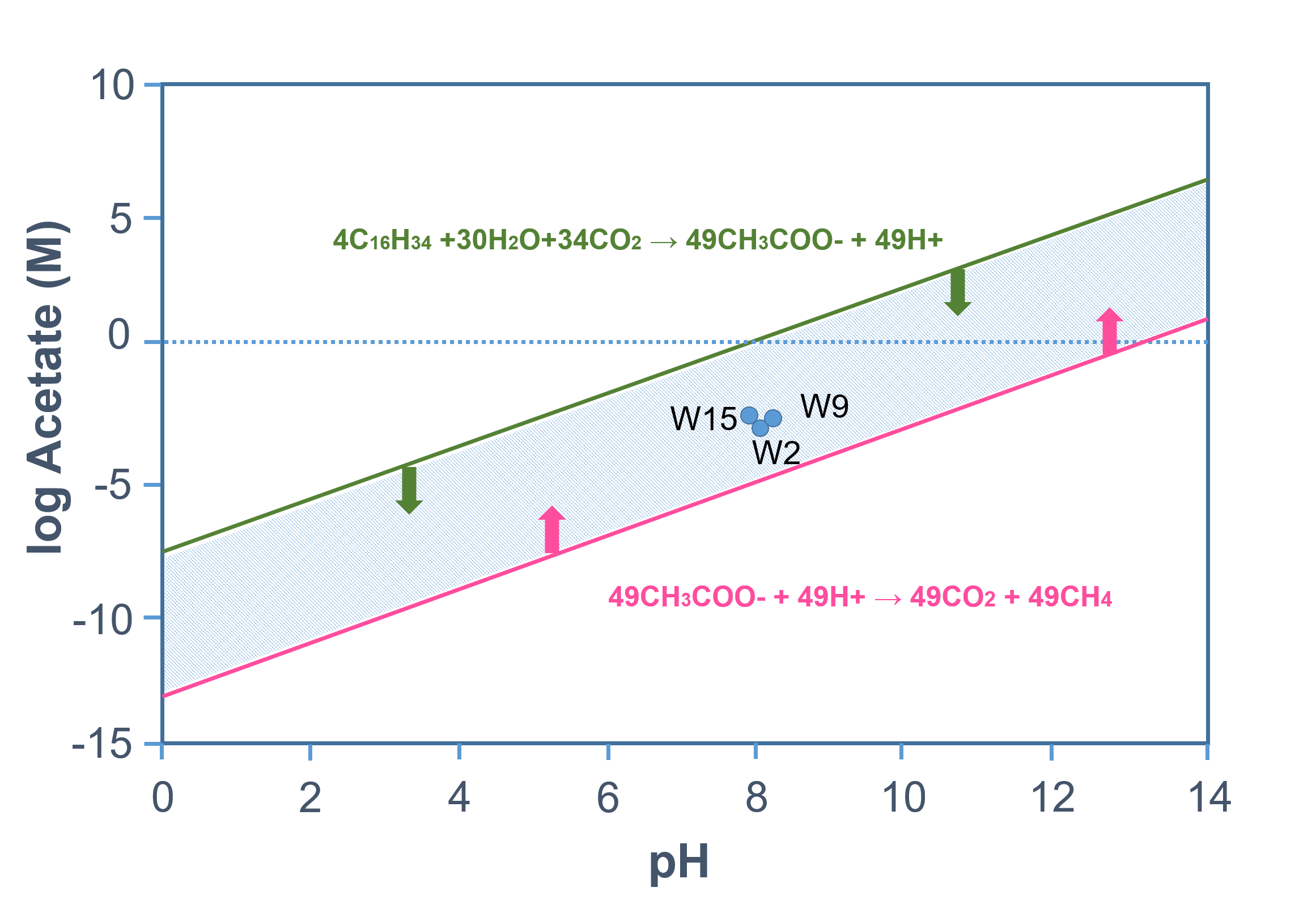

Supplement: Supplementary file 11 — Effect of pH on the range of acetate concentrations when n-alkanes (i.e. hexadecane as representative for calculation) are oxidized to acetate as sole fermentation product and coupled to acetoclastic methanogenesis. The green line represents the oxidation of hexadecane to acetate; red line represents the conversion of acetate to CH4. Blue dots represent acetate concentration and pH values present in wells W2, W9 and W15. The arrows indicate conditions under which the process becomes exergonic. The highlighted zone (light blue) indicates a window of opportunity where oxidization of hexadecane to acetate coupled to acetoclastic methanogenesis is thermodynamically favorable. The figure was modified from [30]. (TIFF 682 kb) [file 40168_2017_392_MOESM11_ESM.tif]
