# Supplementary material for: Metabolic capability and in situ activity of microorganisms in an oil reservoir
Source: Microbiome. 2018 Jan 5;6:5. doi: 10.1186/s40168-017-0392-1 (PMC5756336; doi:10.1186/s40168-017-0392-1)
Supplement: Supplementary file 10 — Thermodynamic constraint of acetate concentration on oxidation of hexadecane to acetate. (DOCX 15 kb) [file 40168_2017_392_MOESM10_ESM.docx]

**Table S7 | Thermodynamic constraint of acetate concentration on oxidation of hexadecane to acetate.**

| Sample | Thermodynamic threshold [1] | W2 | W9 | W15 |
| --- | --- | --- | --- | --- |
| log acetate (M) when pH around 8 | < 0 | -3.4 | -2.9 | -2.7 |
| pH | 8 | 8 | 8.2 | 7.9 |

**Reference**

1. Dolfing, J., Larter, S. R. & Head, I. M. Thermodynamic constraints on methanogenic crude oil biodegradation. *The ISME journal*. 2008;2:442–452.
