# Supplementary material for: Metabolic capability and in situ activity of microorganisms in an oil reservoir
Source: Microbiome. 2018 Jan 5;6:5. doi: 10.1186/s40168-017-0392-1 (PMC5756336; doi:10.1186/s40168-017-0392-1)
Supplement: Supplementary file 12 — Geochemical characterization of production water from three wells from the Jiangsu oil field, China. (* ND, not detected.) (DOCX 15 kb) [file 40168_2017_392_MOESM12_ESM.docx]

**Table S8 | Geochemical characterization of production water from three wells from the Jiangsu oil field, China.**(* ND, not detected.)

| Sample | W2 | W9 | W15 |
| --- | --- | --- | --- |
| Depth (m) | 1499.4 | 1809.5 | 1558.2 |
| Temperature (°C) | 66 | 61 | 76 |
| pH | 8.2 | 8 | 7.9 |
| Oil viscosity (mPa*s) | 14.5 | 26.3 | 36.3 |
| Water content (%) | 91 | 84.8 | 96.9 |
| Total mineralization (total salinity) (g/L) | 22658.3 | 22109.6 | 20081.4 |
| Na (mg/L) | 4143.3 | 8318.8 | 3692.4 |
| K (mg/L) | 200.1 | 11.9 | 4.6 |
| Mg (mg/L) | 11.6 | 24.9 | 3 |
| Ca (mg/L) | 28.17 | 78.4 | 18 |
| NH_4_ (mg/L) | 64.8 | 21.9 | 7.2 |
| PO_4_ (mg/L) | 95.4 | 292.5 | 258.8 |
| Cl (mg/L) | 4034.3 | 12982.7 | 6020.7 |
| SO_4_ (mg/L) | 244 | 307.8 | 45 |
| S_2_O_3_^2-^ (mg/l) | 89.7 | 149.6 | 38.5 |
| HCO_3_ (mg/L) | 3123.3 | 3205.4 | 5735.1 |
| S^2-^ (mg/L) | 9.4 | 9.7 | ND* |
| NO_3_ (mg/L) | 1.5 | 8.5 | 5.7. |
| Acetate (mg/l) | 24.5 | 84.7 | 108.7 |
