# Supplementary material for: Metabolic capability and in situ activity of microorganisms in an oil reservoir
Source: Microbiome. 2018 Jan 5;6:5. doi: 10.1186/s40168-017-0392-1 (PMC5756336; doi:10.1186/s40168-017-0392-1)
Supplement: Supplementary file 13 — Supplementary results and discussion. (DOCX 17 kb) [file 40168_2017_392_MOESM13_ESM.docx]

**Supplementary Results and Discussion**

*Production water analysis*

The geochemical parameters of samples obtained from three oil wells of the Jiangsu oil field (Jiangsu, China) and fluids produced from these wells were analyzed (Supplementary Table S1). All three wells were exhibiting elevated temperatures of over 61°C, which influences the microbial communities present in these wells. Elevated sulfate and thiosulfate concentrations along with the presence of sulfide in wells W2 and W9 suggest the potential for sulfidogenic microorganisms in these two wells. Acetate concentrations (24.5 - 108.7 mg/l) in production water from all wells were similar to previous reports from oil fields undergoing crude oil degradation [1], suggesting an active microbial community in these samples

**Reference**

1. Fisher, J. B. Distribution and occurrence of aliphatic acid anions in deep subsurface waters. Geochimica et Cosmochimica Acta. 1987;51:2459–2468.
