# Supplementary material for: Metabolic capability and in situ activity of microorganisms in an oil reservoir
Source: Microbiome. 2018 Jan 5;6:5. doi: 10.1186/s40168-017-0392-1 (PMC5756336; doi:10.1186/s40168-017-0392-1)
Supplement: Supplementary file 14 — Supplementary materials and methods. (DOCX 29 kb) [file 40168_2017_392_MOESM14_ESM.docx]

**Supplementary Information**

**Supplementary Materials and methods**

*Sample location and geochemical measurements*

The Jiangsu oil field (119.418°N, 32.389°E) is located in Yangzhou, Jiangsu province, China. Temperature was measured at depth where bulk oil is present. Effective porosity and viscosity were measured using produced crude oil. To measure ion contents, 5 mL production water was collected for each sample and centrifuged at 12,000 × g at 4 °C in the lab for 10 min to discard microorganisms. The supernatant was extracted and ion concentrations therein were measured by ion chromatography (ICS1100), which was equipped with an IONPAC AS11-HC column (temperature: 30℃, eluent: KOH gradient elution, detector: conductivity detector and ampere detector, suppressor: anion suppressor), and an IONPAC CS12A column (temperature: room temperature, eluent: 20mM methylsulfonic acid isocratic elution, detector: conductivity detector, suppressor: cation suppressor).

*Sequence assembly*

Since SPAdes v3.7.0 [1] does not handle multiple samples for co-assembly, metagenome datasets of the three samples (W2, W9, and W15) were merged before assembly. Different K-mers (-k 33, 37, 45, 51, 63, 71 --meta) were tested during processing, and contigs with different K-mer used were evaluated using MetaQUAST v4.0 [2]. K-mer was chosen based on long contigs assembly. A second assembly was carried out with this K-mer to generate a scaffold file, which merge contigs with gaps based on pair of reads. These final scaffolds were used for subsequent binning, annotation and as reference for metatranscriptomic analysis.

*Decontamination and evaluation of GBs*

GBs produced from differential binning step were uploaded to ProDeGe server [3], and clean scaffolds were determined based on homology and feature-based methodologies. GBs with clean scaffolds were downloaded and completeness and contamination of clean GBs were evaluated using lineage-specific marker genes.

*Annotation*

Nucleotide sequence files of GBs were uploaded to RAST [4] for annotation. Next, CDS of GBs were found locally using Prodigal (v2.50) with default parameters, and amino acid sequence files were obtained at the same time. These amino acid sequence files were submitted to BlastKOALA [5] in KEGG [6] for determining KEGG orthologies and assign KO numbers to these orthologies. With this method, both system annotations and KO numbers for genes of GBs were obtained. In addition, result of HMM search against the co-assembly file were used to help curate features that are associated with hydrocarbon degradation in *Archaeoglobus*-like Bin9, *Acinetobacter*-like Bin1 and *Pseudomona*s-like Bin19.

*Construction of genome tree*

Amino acid sequences of GBs were obtained using Prodigal (v2.50) [7] with default parameters. Based on the information of ‘closest neighbor’ function in RAST, the proteins sequences of the closest reference strains of GBs were downloaded from Genebank. All reference genomes and GBs were combined and a genome tree was constructed using Phylophlan v0.99 with default settings [8].

*Construction of AssA phylogenetic tree*

Reference amino acid sequences of AssA downloaded from NCBI website. Reference sequences and amino acid sequence of putative AssA in Bin9 were aligned using MAFFT [9] with iterative refinement methods ‘G-INS-i’. Conserved positions of alignments were kept on Gblock Server (<http://molevol.cmima.csic.es/castresana/Gblocks_server.html>) using less stringent selection settings [10]. Neighbor-joining phylogenetic tree was constructed in MEGA 7.0 [11], and bootstrap support values were determined with non-parametric bootstrapping (1000 replicates).

**Reference**

1. Bankevich A, Nurk S, Antipov D, Gurevich A a., Dvorkin M, Kulikov AS, et al. SPAdes: A New Genome Assembly Algorithm and Its Applications to Single-Cell Sequencing. Journal of Computational Biology. 2012;19:455–77.

2. Mikheenko A, Saveliev V, Gurevich A. MetaQUAST: Evaluation of metagenome assemblies. Bioinformatics. 2016;32:1088–90.

3. Tennessen K, Andersen E, Clingenpeel S, Rinke C, Lundberg DS, Han J, et al. ProDeGe: a computational protocol for fully automated decontamination of genomes. The ISME Journal [Internet]. Nature Publishing Group; 2015;10:1–4. Available from: http://www.nature.com/doifinder/10.1038/ismej.2015.100

4. Aziz RK, Bartels D, Best AA, DeJongh M, Disz T, Edwards RA, et al. The RAST Server: rapid annotations using subsystems technology. BMC genomics [Internet]. 2008;9:75. Available from: http://bmcgenomics.biomedcentral.com/articles/10.1186/1471-2164-9-75

5. Kanehisa M, Sato Y, Morishima K. BlastKOALA and GhostKOALA: KEGG Tools for Functional Characterization of Genome and Metagenome Sequences. Journal of Molecular Biology [Internet]. The Authors; 2016;428:726–31. Available from: http://dx.doi.org/10.1016/j.jmb.2015.11.006

6. Ogata H, Goto S, Sato K, Fujibuchi W, Bono H, Kanehisa M. KEGG: Kyoto encyclopedia of genes and genomes. Nucleic Acids Research. 1999;27:29–34.

7. Hyatt D, Locascio PF, Hauser LJ, Uberbacher EC. Gene and translation initiation site prediction in metagenomic sequences. Bioinformatics. 2012;28:2223–30.

8. Segata N, Börnigen D, Morgan XC, Huttenhower C. PhyloPhlAn is a new method for improved phylogenetic and taxonomic placement of microbes. Nature communications [Internet]. 2013;4:2304. Available from: http://www.pubmedcentral.nih.gov/articlerender.fcgi?artid=3760377&tool=pmcentrez&rendertype=abstract

9. Yamada KD, Tomii K, Katoh K. Application of the MAFFT sequence alignment program to large data - reexamination of the usefulness of chained guide trees. Bioinformatics (Oxford, England) [Internet]. 2016;1–6. Available from: http://www.ncbi.nlm.nih.gov/pubmed/27378296

10. Talavera G, Castresana J. Improvement of phylogenies after removing divergent and ambiguously aligned blocks from protein sequence alignments. Systematic Biology. 2007;56:564–77.

11. Kumar S, Stecher G, Tamura K. MEGA7: Molecular Evolutionary Genetics Analysis version 7.0 for bigger datasets. Molecular biology and evolution [Internet]. 2016;33:msw054. Available from: http://www.ncbi.nlm.nih.gov/pubmed/27004904
